# Substituent Effects of Adamantyl Group on Amido Ligand in Syndiospecific Polymerization of Propylene with *Ansa*-Dimethylsilylene(Fluorenyl)(Amido) Zirconium Complex

**DOI:** 10.3390/polym9110632

**Published:** 2017-11-21

**Authors:** Yanjie Sun, Shuhui Li, Takeshi Shiono, Zhengguo Cai

**Affiliations:** 1 State Key Laboratory for Modification of Chemical Fibers and Polymer Materials, College of Material Science and Engineering, Donghua University, Shanghai 201620, China; syj-178@163.com (Y.S.); 15000855167@163.com (S.L.); 2 Graduate School of Engineering, Hiroshima University, Higashi-Hiroshima 739-8527, Japan

**Keywords:** constrained geometry catalysts, zirconium complex, adamantyl substituent, propylene polymerization, syndiospecificity

## Abstract

A series of new fluorenylamido-ligated zirconium complexes bearing an electron-donating adamantyl group on the amido ligand were synthesized and characterized by elemental analysis, ^1^H NMR, and single crystal X-ray analysis. The coordination mode of the fluorenyl ligand to the zirconium metal was *η*^3^ manner, and all the complexes were *C_s_*-symmetric in solution. The complexes showed moderate activity (1.0 × 10^5^ g-polymer mol-Zr^−1^·h^−1^), even at a low Al/Zr ratio of 50. The increase of propylene pressure improved the activity by one order of magnitude (up to 1.0 × 10^6^ g-polymer mol-Ti^−1^·h^−1^). All catalyst systems gave syndiotactic polypropylene, where the complex containing the 3,6-di-*t*-butyl fluorenyl ligand was more effective for the enhancement of the syndiospecificity. The increase of propylene pressure also improved the syndiospecificity with the syndiotactic pentad of 0.96 and the melting point of 159 °C.

## 1. Introduction

Developments of single-site catalysts based on group-4 metallocenes demonstrated a well-defined mechanism for the relationship between the symmetry of the complex and the stereospecificity, in which a little change of the ligand dramatically influenced the polymerization performances, such as the activity, stereospecificity, and molecular weight [1,2,3]. Ewen et al. first reported the preparation of syndiotactic polypropylene (*syn*-PP) by using *C*_s_-symmetric metallocene in 1988 [4]. Since this report, much effort has been made towards achieving the synthesis of *syn*-PP with single-site catalysts, including doubly bridged metallocenes [5], constrained geometry catalysts (CGCs) [6], and nonmetallocene catalysts [7,8,9,10,11,12]. Among these catalysts, group-4 CGCs have attracted much attention for their capabilities of improving copolymerization ability [13,14,15,16,17,18,19,20,21,22], stereospecificity [23,24,25,26,27], and living polymerization characteristics [28,29]. Many attempts have been made to improve the catalytic performance of CGC catalysts by changing the electronic and steric properties of the ligand.

**Figure 1:**
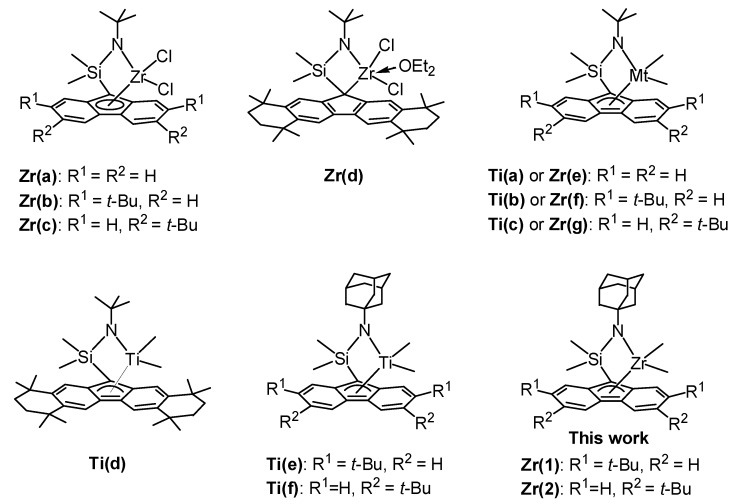
Structures of *ansa*-(fluorenyl)(amido)-ligated complexes.

Razavi et al. reported that the introduction of *t-*butyl substituent on the fluorenyl ligand of [Me_2_Si(*t*-Bu-N)(di-*t*-Bu-Flu)ZrCl_2_] (**Zr(a**–**c)**, Figure 1) improved both the activity and *syn*-specificity with *syn*-pentad (rrrr) of 0.87 [23,24]. Miller et al. demonstrated that sterically expanded zirconium complex (**Zr(d)**, Figure 1) combined with methylaluminoxane (MAO) was strikingly active to give *syn*-PP with unsurpassed *syn*-specificity (rrrr > 0.99) and melting temperature (*T*_m_ up to 165 °C) [25]. The results indicated that alkyl substituents on the fluorenyl ligand of zirconium complexes play an important role in catalytic activity and *syn*-specificity. However, the electronic effect of the amido ligand was not investigated.

We previously synthesized the corresponding dimethyltitanium complexes (**Ti(a**–**c)**, Figure 1) and found that the introduction of *t*-butyl group on the 3,6-position of the fluorenyl ligand improved both the activity and *syn*-specificity in the living polymerization of propylene [30]. However, sterically expanded titanium complex (**Ti(d)**, Figure 1) showed low *syn*-specificity, which differed from that of the corresponding zirconium complex **Zr(d)** [31]. On the other hand, dimethylzirconium complexes [Me_2_Si(*t*-Bu-N)(di-*t*-Bu-Flu)ZrMe_2_] (**Zr(e**–**g)**, Figure 1) showed very low activity for propylene polymerization [32]. Recently, we reported that the introduction of an electron-donating adamantyl substituent on the amido ligand of dimethyltitanium complexes (**Ti(e**–**f)**, Figure 1) exhibited remarkably high activity with an Al/Ti ratio of 20 without changing *syn*-specificity or livingness [33,34]. In this paper, we synthesized dimethylzirconium complexes (**Zr(1)** and **Zr(2)**, Figure 1) by using the same ligand to investigate the substitute effects of the adamantyl group on the amido ligand of fluorenylamido-ligated zirconium complexes in propylene polymerization.

## 2. Experimental Section

### 2.1. Materials

All operations were carried out under N_2_ by using standard Schlenk techniques, and all solvents were purified by a PS-MD-5 solvent purification system (Innovative Technology (China) Ltd., Hong Kong, China). A research grade propylene was purified by being passed through a dehydration column of ZHD-20 and a deoxidation column of ZHD-20A before use. Modified methylaluminoxane (MMAO) was donated by Tosoh-Finechem Co. (Shunan, Japan). The ligands and zirconium complexes were prepared according to the procedure reported in the literature [32,33].

### 2.2. Synthesis of Complexes

#### 2.2.1. Synthesis of [(1-Adamantyl)NSiMe_2_(2,7-di-*t*-BuFlu)]ZrMe_2_ (**Zr(1)**)

MeLi (1.6 M in ether 10.5 mL, 16.8 mmol) was added dropwise at −20 °C to a solution of ligand (2,7-di-*t*-BuFlu)SiMe_2_(1-Adamantyl) (1.94 g, 4.0 mmol) in 60 mL of diethylether. The resultant orange solution was stirred at room temperature for 4 h. To a solution of ZrCl_4_ (0.93 g, 4.0 mmol) in 30 mL pentane, the diethylether solution of the lithium salt was added, which gave a yellow suspension. After stirring for 12 h, the solvent was removed and the residue was extracted with hexane. Then the hexane solution was concentrated and cooled at −30 °C to yield **Zr(1)** as yellow crystals (0.72 g, 1.24 mmol, 31% yield).

^1^H NMR (CDCl_3_) (Figure S1): δ = 8.00 (d, 2H, Flu); 7.73 (s, 2H, Flu); 7.45 (dd, 2H, Flu); 2.07 (s, 3H, Ad); 1.80(d, 6H, Ad); 1.64 (d, 6H, Ad); 1.41 (s, 18H, *t*-Bu-Flu); 0.84 (s, 6H, SiCH_3_); −0.11 (s, 6H, ZrCH_3_). ^13^C NMR (CDCl_3_) (Figure S2): 150.8 (Flu); 136.0 (Flu); 122.9 (Flu); 122.4 (Flu); 122.1 (Flu); 120.5 (Flu); 56.1 (Flu); 48.0 (Zr-(CH_3_)_2_); 39.6 (Ad); 36.5 (Ad); 35.4 (Flu-(C(CH_3_)_3_)_2_); 31.8 (Ad); 31.5 (Flu-(C(CH_3_)_3_)_2_); 30.3 (Ad); 7.2 (Si-(CH_3_)_2_). Elemental analysis for C_35_H_51_NSiZr (calc/found, %): C, 69.47/69.26; H, 8.50/8.41; N, 2.31/2.26.

#### 2.2.2. Synthesis of [(1-Adamantyl)NSiMe_2_(3,6-di-*t*-BuFlu)]ZrMe_2_ (**Zr(2)**)

Complex **Zr(2)** was synthesized in a method similar to that for **Zr(1)**, and yellow crystals were obtained in 33% yield.

^1^H NMR (CDCl_3_) (Figure S3): δ = 8.06 (s, 2H, Flu); 7.70 (d, 2H, Flu); 7.46 (dd, 2H, Flu); 2.06 (s, 3H, Ad); 1.82 (d, 6H, Ad); 1.64 (d, 6H, Ad); 1.46 (s, 18H, *t*-Bu-Flu); 0.82 (s, 6H, SiCH_3_); −1.05 (s, 6H, ZrCH_3_). ^13^C NMR (CDCl_3_) (Figure S4): 147.1 (Flu); 134.0 (Flu); 127.2 (Flu); 124.7 (Flu); 124.4 (Flu); 118.3 (Flu); 56.1 (Flu); 48.1 (Zr-(CH_3_)_2_); 39.6 (Ad); 36.5 (Ad); 35.1 (Flu-(C(CH_3_)_3_)_2_); 32.0 (Ad); 31.9 (Flu-(C(CH_3_)_3_)_2_); 30.3(Ad); 7.0 (Si-(CH_3_)_2_). Elemental analysis for C_35_H_51_NSiZr (calc/found, %): C, 69.47/69.38; H, 8.50/8.46; N, 2.31/2.29.

### 2.3. Polymerization Procedure

Atmospheric polymerization of propylene was performed in a 100-mL glass reactor equipped with a magnetic stirrer and carried out according to the semi-batch method. At first, the reactor was charged with prescribed amounts of MMAO/2,6-di-*tert*-butyl-4-methyl phenol (BHT) or dired MMAO (dMMAO) and solvent (heptane). After the solution of the cocatalyst was saturated with gaseous propylene under atmospheric pressure, polymerization was started by the addition of 1 mL solution of the zirconium complex in heptane, and the consumption rate of propylene was monitored by a mass flow meter.

High pressure polymerization of propylene was performed in a 200-mL Quick-Open Micro Autoclaves/Pressure Vessel purchased from Anhui Kemi Machinery Technology Co., Ltd. (Hefei, China) Before polymerization, the reactor was cleaned and evacuated at 110 °C for 1 h. Certain amounts of the MMAO/BHT, heptane were added into the reactor under a nitrogen atmosphere, and the mixture was stirred continuously. When the temperature was established, the catalyst solution of heptane was added into the reactor. The reactor was then pressurized with propylene. The polymerization was conducted for a certain time, and terminated with acidic alcohol. The polymers obtained were washed by alcohol to remove MMAO and ligand residue, and dried under vacuum at 80 °C for 6 h until a constant weight was reached.

### 2.4. Analytical Procedure

The single crystals were mounted under a nitrogen atmosphere at a low temperature, and data collection was made on a Bruker APEX2 diffractometer (Bruker, Karlsruhe, Germany) using graphite monochromated with Mo Ka radiation (=0.71073 Å). The SMART program package (University of Göttingen, Göttingen, Germany) was used to determine the unit cell parameters. The absorption correction was applied using the SADABS program (University of Göttingen, Göttingen, Germany) [35]. All structures were solved by direct methods and refined on *F*^2^ by full-matrix least-squares techniques with anisotropic thermal parameters for non-hydrogen atoms. Hydrogen atoms were placed at calculated positions and were included in the structure calculation. Calculations were carried out using the SHELXS-97, SHELXL-2014, or Olex2 program (Bruker AXS Inc., Madison, WI, USA) [36,37,38,39,40,41]. Crystallographic data are summarized in Table 1.

Molecular weights and molecular weight distributions of polymers were measured by a polymer laboratory PL GPC-220 chromatograph (Agilen, Santa Clara, CA, USA) equipped with one PL1110-1120 column and two PL MIXED-B 7.5 × 300 mm columns at 150 °C using 1,2,4-trichlorobenzene as a solvent. The parameters for universal calibration were *K* = 7.36 × 10^−5^, α = 0.75 for polystyrene standard and *K* = 1.03 × 10^−4^, α = 0.78 for PP samples. Differential scanning calorimeter (DSC) analyses were performed on a TA Q2000 instrument (Waters, New Castle, DE, USA) and the DSC curves of the samples were recorded under a nitrogen atmosphere at a heating rate of 10 °C/min from 40 to 200 °C. The ^1^H NMR spectra of complexes were recorded and the ^13^C NMR spectra of PPs were measured on a Bruker Asend™ 600 spectrometer (Bruker, Karlsruhe, Germany). The chemical shifts of the ^1^H NMR spectra were referenced to the residual proton resonance of chloroform-*d* (δ: 7.26), and the ^13^C NMR spectra of PPs were recorded at 110 °C and referenced to the resonance of 1,1,2,2-tetrachloroethane-*d*_2_ (δ: 74.47).

## 3. Results and Discussion

### 3.1. Molecular Structure of Complexes

**Figure 2:**
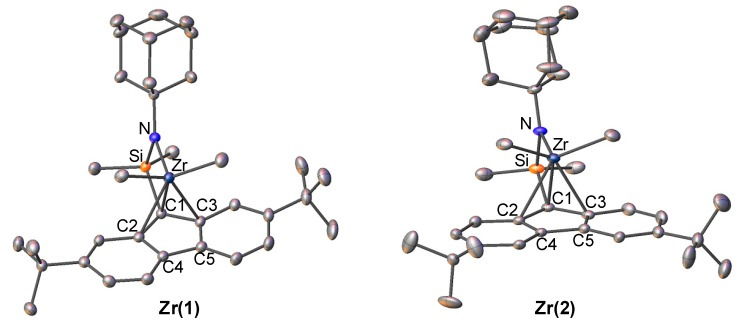
Structure of fluorenylamidotitanium complexes **Zr(1)** and **Zr(2)**. Hydrogen atoms are omitted for clarity. Atoms are drawn at the 40% probability level.

The zirconium complexes were synthesized by a one-pot reaction of the corresponding ligand with 4 equivalent of methyl lithium and 1 equiv of ZrCl_4_. ^1^H NMR spectrum of the methyl groups bonded to Zr and Si atoms in both zirconium complexes indicated that the complexes exhibited a *C*_s_-symmetric nature in solution, respectively. The molecular structures of **Zr(1)** and **Zr(2)** were characterized by single crystal X-ray analysis. The structures are shown in Figure 2, and the selected bond lengths and angles of complexes are shown in Table 2. The lengths between the zirconium metal and five-member carbons of fluorenyl ligand in **Zr(1)** and **Zr(2)** are very close to those previously reported for *t*-butyl amido complex (**Zr(f)**), in which the fluorenyl ligand was coordinated to the zirconium in an *η*^3^ manner. We applied Tolman cone angles of the amino ligand (θ value in Table 1) to evaluate the steric effect of the amido ligand [42]. The θ values of **Zr(1)** and **Zr(2)** were 71.06 and 68.80°, respectively, which were close to that of the previously reported **Zr(f)** (70.12°). These results indicated that *t*-butyl and 1-admantyl groups possess a similar steric environment around the cationic zirconium metal, thus maintaining the hapticity of the fluorenyl ligand. We therefore can investigate the true electronic effect of the adamantyl substituent on the amido ligand with these fluorenylamido-ligated zirconium complexes for propylene polymerization.

### 3.2. Propylene Polymerization

Propylene polymerizations were performed by **Zr(1)** and **Zr(2)** activated with trialkylaluminum-free dried MMAO (dMMAO) under atmospheric pressure of propylene in heptane at 0 and 20 °C, and the results are summarized in Table 3. For comparison, the same polymerization conditions as those previously reported for propylene polymerization using **Zr(f)** and **Zr(g)** were employed [32]. The complexes **Zr(f)** and **Zr(g)** conducted propylene polymerization at 20 °C, although they did not show any activity at 0 °C. On the other hand, **Zr(1)** and **Zr(2)** carried out propylene polymerization with moderate activity of ~1.2 × 10^5^ g-polymer mol-Zr^−1^·h^−1^ to produce low molecular weight PP, even at 0 °C. The activities of **Zr(1)** and **Zr(2)** were higher than those of **Zr(f)** and **Zr(g)** at 20 °C. The results testified that the electronic effect of the adamantyl group on the amido ligand plays an important role in this catalyst system rather than having a steric effect, since **Zr(1)**, **Zr(2)**, and **Zr(g)** possess a similar steric environment around the amido ligand. The high performance of **Zr(1)** and **Zr(2)** can be ascribed to the decreased electrophilicity of the zirconium cation caused by the electron-donating adamantyl group [43], which enhances the separation of the counter anion.

The use of the modification of trialkylaluminum in MMAO with 2,6-di-*tert*-butyl-4-methyl phenol (BHT) as the cocatalyst [44,45] resulted in levels of activity approximately twice as high (up to 2.1 × 10^5^ g-polymer mol-Zr^−1^·h^−1^). The same phenomenon was also observed in propylene polymerization with fluorenylamido-ligated dimethyl titanium catalysts (**Ti(e**–**f)**), where the presence of *i*Bu_2_Al(OC_6_H_2_*t*Bu_2_Me) derived from the reaction of *i*Bu_3_Al and BHT promoted the efficient separation of the active ion pair to improve the activity [33].

The high cost of single-site catalysts, owing to the requirement of a very high Al/metal ratio to achieve high activity, is a serious limitation for industrial applications. The effect of the Al/Zr ratio in these catalysts was thus investigated (entries 9–12). Although the activity decreased according to the decrease of the Al/Zr ratio from 400 to 100, complex **Zr(1)** still showed moderate activity of 1.0 × 10^5^ g-polymer mol-Zr^−1^·h^−1^ even with an Al/Zr ratio of 50 (entry 12), the value of which was comparable to those of **Zr(1)** and **Zr(2)** activated by dMMAO with Al/Ti = 400.

The increase of propylene pressure from 1.0 to 8.0 atm resulted in the increase of the activity by one order of magnitude (up to ~1.0 × 10^6^ g-polymer mol-Ti^−1^ h^−1^, entries 13 and 14). We previously reported that the propagation rate of propylene polymerization with **Ti(a**–**c)**–dMMAO at 0 °C was increased linearly against the propylene pressure [46].

The melting temperatures of the PPs obtained are also shown in Table 2. All of the catalyst systems gave crystalline polymers with high *T*_m_ values. The PP obtained with **Zr(2)** showed a higher *T*_m_ value than that obtained with **Zr(1)** in the same polymerization conditions, and the *T*_m_ value slightly increased with the increase of the propylene pressure in each catalyst system. A higher *T*_m_ value should be ascribed to the higher *syn*-tacticity of PP.

The steric pentad distributions calculated by the ^13^C NMR spectra (Figures S5 and S6) of the methyl region of PPs are shown in Table 4. The results indicated that the PPs obtained were syndiotactic with high rrrr value, and the PP obtained by **Zr(2)** showed a higher rrrr value of 0.96. The *syn*-specific polymerization was conducted via an enantiomorphic site-controlled mechanism with a *C*_s_-symmetric catalyst. In this system, two types of stereodefects are present: one is rmrr arising from the chain migration without monomer insertion, and the other is rmmr arising from the monomer mis-insertion [4]. Both rmrr and rmmr values were decreased in the following order: **Zr(1)** (0.026) > **Zr(2)** (0.003) and **Zr(1)** (0.021) > **Zr(2)** (0.007). These results indicated that the *t*-butyl groups at the 3,6-position of the fluorenyl ligand effectively improve the chain migration and enantioselectivity of the propylene monomer. This result is in agreement with that of propylene polymerization with **Ti(b**–**c)**–dMMAO, where the 3,6-position was more effective than the 2,7-position in improving *syn*-specificity [30].

## 4. Conclusions

The substituent effects of an electron-donating adamantyl group on the amido ligand of fluorenylamido-ligated zirconium catalysts were investigated. Complexes **Zr(1)** and **Zr(2)** showed moderate activity of ~1.0 × 10^5^ g-polymer mol-Zr^−1^·h^−1^ with a low Al/Ti ratio of 50. Complex **Zr(2)** containing 3,6-di-*t*-butyl fluorenyl ligand and the increase of propylene pressure were effective for the improvement of polymerization activity (up to 1.0 × 10^6^ g-polymer mol-Zr^−1^·h^−1^) and *syn*-specificity to produce a highly *syn*-tactic PP with an rrrr value of 0.96 and a melting point of 159 °C. These results are in good agreement with the substituent effects of the adamantyl group on the amido ligand of the fluorenylamido-ligated titanium complex.

## Figures and Tables

**Table 1:** Crystallographic data and parameters for **Zr(1)** and **Zr(2)**.

Complex	Zr(1)	Zr(2)
**Formula**	C_35_H_51_NSiZr	C_35_H_51_NSiZr
**Formula weight**	605.07	605.07
**Crystal system**	Triclinic	Monoclinic
**Space group**	*P*$\bar{1}$	*P*1 2_1_/*c*1
***a* (Å)**	10.5309(10)	12.673(3)
***b* (Å)**	11.6688(11)	19.886(4)
***c* (Å)**	14.6617(14)	14.198(3)
***Β* (deg)**	75.818(2)	111.899(4)
***V* (Å^3^)**	1584.3(3)	3320.0(12)
***Z***	2	4
***F*(000)**	644	1288
**Dcalcd. (g.cm^−3^)**	1268	1211
**μ (mm^−1^)**	0.408	0.390
**Theta range for data collection**	1.924 to 30.715°	1.842 to 25.499°
**Reflections collected**	16,281	23,033
**Independent reflections**	9719 [*R*(int) = 0.2619]	6155 [*R*(int) = 0.0635]
**Final *R* indices [*I* > 2δ*(I*)]**	*R1* = 0.0387, *wR2* = 0.0905	*R1* = 0.0546, *wR2* = 0.1417

**Table 2:** Selected bond lengths (Å) and bond angles (degrees) for related complexes.

Parameters	Zr(1)	Zr(2)	Zr(f)
Zr(1)-C(1)	2.3960(17)	2.402(5)	2.385(3)
Zr(1)-C(2)	2.5443(17)	2.535(5)	2.563(3)
Zr(1)-C(3)	2.4948(17)	2.524(5)	2.486(3)
Zr(1)-C(4)	2.6871(17)	2.664(4)	2.702(3)
Zr(1)-C(5)	2.6706(17)	2.655(4)	2.667(3)
Zr(1)-N(1)	2.0536(15)	2.061(4)	2.058(3)
Zr(1)-Si(1)	2.9867(6)	3.005(16)	2.981(3)
θ ^a^ = 2/3 (θ_1_ + θ_2_ + θ_3_)	71.06	68.80	70.12

^a^ Tolman cone angle of amido groups.

**Table 3:** Results of propylene polymerization with zirconium complexes ^a^.

Entry	cat.	Cocatalyst	Al/Zr	P (atm)	Temp (°C)	Time (min)	Yield (g)	Activity ^d^ (×10^3^)	*M*_n_^e^ (×10^3^)	MWD ^e^	*T*_m_^f^ (°C)
1	**Zr(1)**	dMMAO	400	1	0	30	1.29	129	4.4	1.51	139
2	**Zr(2)**	dMMAO	400	1	0	30	1.17	117	3.8	1.42	155
3	**Zr(1)**	dMMAO	400	1	20	30	3.23	323	2.5	1.67	132
4	**Zr(2)**	dMMAO	400	1	20	30	3.56	356	3.1	1.53	148
5 ^b^	**Zr(f)**	dMMAO	400	1	0	30	0	0	-	-	-
6 ^b^	**Zr(g)**	dMMAO	400	1	0	30	0	0	-	-	-
7 ^b^	**Zr(f)**	dMMAO	400	1	20	30	1.77	177	1.17	1.43	125
8 ^b^	**Zr(g)**	dMMAO	400	1	20	30	1.39	139	1.39	1.67	145
9	**Zr(1)**	MMAO/BHT	400	1	0	30	2.12	212	5.2	1.55	139
10	**Zr(1)**	MMAO/BHT	200	1	0	30	1.52	152	4.8	1.43	138
11	**Zr(1)**	MMAO/BHT	100	1	0	30	0.99	99	4.1	1.5	138
12	**Zr(1)**	MMAO/BHT	50	1	0	30	0.95	95	3.8	1.47	138
13 **^c^**	**Zr(1)**	MMAO/BHT	400	8	0	8	1.2	900	7.4	1.81	142
14 **^c^**	**Zr(2)**	MMAO/BHT	400	8	0	8	1.35	1012	2.8	2.32	159

^a^ Polymerization conditions: Heptane = 30 mL, Zr = 20 μmol, propylene = 1 atm; ^b^ Data taken from Reference [32]; ^c^ Zr = 10 μmol; ^d^ Activity in g of PP/(mol of Ti.h); ^e^ Number-average molecular weight and molecular weight distribution determined by gel-permeation chromatography GPC using universal calibration; ^f^ Melting points determined by DSC.

**Table 4:** Steric pentad distributions for samples in Table 3 (entries 13 and 14) ^a^.

Catalyst	Stereosequence Distribution ^a^								
	mmmm	mmmr	rmmr	mmrr	mmrm + rmrr	rmrm	rrrr	mrrr	mrrm
**Zr(1)**	0.00	0.00	0.021	0.043	0.026	0.021	0.820	0.069	0.00
**Zr(2)**	0.00	0.00	0.007	0.014	0.003	0.00	0.958	0.018	0.00

^a^ Determined by ^13^C NMR spectroscopy.

## Supplementary Materials

The following are available online at www.mdpi.com/2073-4360/9/11/632/s1, Figure S1: ^1^H NMR spectrum of complex **Zr(1)**, Figure S2: ^13^C NMR spectrum of complex **Zr(1)**, Figure S3: ^1^H NMR spectrum of complex **Zr(2)**, Figure S4: ^13^C NMR spectrum of complex **Zr(2)**, Figure S5: ^13^C HMR spectrum of the methyl region of polypropylene obtained with **Zr(1)** (entry 13, Table 3), Figure S6: ^13^C HMR spectrum of the methyl region of polypropylene obtained with **Zr(2)** (entry 14, Table 3).
