# Supplementary material for: Substituent Effects of Adamantyl Group on Amido Ligand in Syndiospecific Polymerization of Propylene with Ansa-Dimethylsilylene(Fluorenyl)(Amido) Zirconium Complex
Source: Polymers (Basel). 2017 Nov 21;9(11):632. doi: 10.3390/polym9110632 (PMC6418878; doi:10.3390/polym9110632)
Supplement: Supplementary file 1 [file polymers-09-00632-s001.zip › polymers-239760-supplementary/polymers-239760-supplementary.doc]

# Supporting Information

**Substituent effects of adamantyl group on amido ligand in syndiospecific polymerization of propylene with *ansa*-dimethylsilylene(fluorenyl)(amido) zirconium complex**

Yanjie Sun^1^, Shuhui Li^1^, Takeshi Shiono^2*^, Zhengguo Cai^1*^

# ^1^State Key Laboratory for Modification of Chemical Fibers and Polymer Materials, College of Material Science and Engineering, Donghua University, Shanghai 201620, China;

# ^2^ Graduate School of Engineering, Hiroshima University, Higashi-Hiroshima, 739-8527, Japan

# * E-Mail: [**tshiono@hiroshima-u.ac.jp**](mailto:tshiono@hiroshima-u.ac.jp) (S.T.); E-Mail: [**caizg@dhu.edu.cn**](mailto:caizg@dhu.edu.cn) (Z.C.); Tel./Fax: +86-21-67792453 (Z.C.)

**Figure S1.** ^1^H NMR spectrum of complex **Zr(1)**.

**Figure S2.** ^13^C NMR spectrum of complex **Zr(1)**.

**Figure S3.** ^1^H NMR spectrum of complex **Zr(2)**.

**Figure S4.** ^13^C NMR spectrum of complex **Zr(2)**.

**Figure S5.** ^13^C HMR spectrum of the methyl region of polypropylene obtained with **Zr(1)** (entry 13, Table 3).

**Figure S6.** ^13^C HMR spectrum of the methyl region of polypropylene obtained with **Zr(2)** (entry 14, Table 3).
